# In Reply to Hsu et al

**DOI:** 10.1016/j.adro.2025.101946

**Published:** 2026-06-12

**Authors:** Ilker Sengul, Demet Sengul

**Affiliations:** a Division of Endocrine Surgery, Giresun University Faculty of Medicine, Giresun, Turkey; b Department of General Surgery, Giresun University Faculty of Medicine, Giresun, Turkey; c Department of Pathology, Giresun University Faculty of Medicine, Giresun, Turkey

*Nulla dies sine linea.* We sincerely thank our colleagues, Dennstädt et al,^1^ for their thoughtful and prompt reply,^2^ published in volume 10, *Advances in Radiation Oncology,* to our previous letter,^3^ entitled “In Reply to Sengul I and Sengul D,” published in the same volume of *Adv Radiat Oncol*. Their original study, published in the 9th volume of *Adv Radiat Oncol,* provided valuable initial insights into the potential of large language models (LLMs) in highly specialized medical fields, such as radiation oncology. We particularly appreciate their acknowledgment of the rapid evolution of LLMs since the release of ChatGPT in November 2022, a point underscored by the significant advancements seen even in the short period following their initial exploratory investigation.^1^ Dennstädt et al’s^3^ reply effectively addressed the complexities of evaluating LLM performance in medicine by highlighting the trade-offs between multiple-choice questions, which offered clear evaluability, and open-ended questions, which better reflect clinical reality but posed challenges in objective assessment. We concur that clinical questions rarely have a single, unambiguously correct answer, making physician-based evaluations crucial despite inherent subjective factors and varying interrater agreement. Their recent follow-up study, which compared the performance of a medically fine-tuned open-source LLM, Llama3-OpenBioLLM-70B, to human specialists with over 10 years of experience on real-world radiotherapeutic questions, marks a significant step forward. However, this advancement also prompts further critical questions for the ongoing research on LLMs in health care: (1) Given the laborious nature of studies involving real-world clinical questions and human expert comparisons, what methodologies can ensure the reproducibility and generalizability of such findings across diverse clinical settings, patient populations, and different LLM architectures? (2) While LLMs may achieve “expert doctor level” accuracy on medical examination questions and provide helpful answers, how do we systematically evaluate their performance on complex, nuanced, or atypical clinical cases where human reasoning, clinical experience, and ethical considerations extend beyond factual knowledge retrieval? (3) Because LLMs become more integrated into clinical practice, even in an assistive capacity, the “black box” nature of some models and the potential for “hallucinations”—generating incorrect statements that appear evidence-based—remain significant concerns for patient safety. What transparency mechanisms and safeguards are essential for building trust and ensuring responsible integration, especially as models are fine-tuned or combined with explicit knowledge bases? (4) Ultimately, the challenge of evaluating LLM performance without full transparency about their training data persists. Because LLMs are anticipated to have a significant impact on the practice and future of medicine and radiation oncology, collaborative efforts between LLM developers and medical professionals will be crucial to navigating these complex ethical, safety, and integration challenges responsibly.^1^, ^2^, ^3^ The continued discussion and research exemplified by these exchanges are crucial for understanding the true potential and inherent limitations of LLMs because they increasingly impact health care, influencing both clinicians and patients seeking medical information. This issue merits further investigation. We thank Dennstädt et al^1^ for their prompt reply in *Advances Radiation Oncology—bonum commune communitatis.*

## Disclosures

The authors declare no competing interests.
